# Pilot Study of Novel Intermittent Fasting Effects on Metabolomic and Trimethylamine *N*-oxide Changes During 24-hour Water-Only Fasting in the FEELGOOD Trial

**DOI:** 10.3390/nu11020246

**Published:** 2019-01-23

**Authors:** Rachel L. Washburn, James E. Cox, Joseph B. Muhlestein, Heidi T. May, John F. Carlquist, Viet T. Le, Jeffrey L. Anderson, Benjamin D. Horne

**Affiliations:** 1 Texas Tech University Health Sciences Center, Lubbock, TX 79415, USA; rachel.washburn@ttuhsc.edu; 2 Department of Biochemistry, University of Utah, Salt Lake City, UT 84132, USA; jcox@cores.utah.edu; 3 Intermountain Medical Center Heart Institute, Cardiology Division, Department of Internal Medicine, University of Utah, Salt Lake City, UT 84107, USA; jbrent.muhlestein@imail.org (J.B.M.); john.carlquist@imail.org (J.F.C.); jeffreyl.anderson@imail.org (J.L.A.); 4 Intermountain Medical Center Heart Institute, Salt Lake City, UT 84107, USA; heidi.may@imail.org; 5 Intermountain Medical Center Heart Institute, Rocky Mountain University of Health Professions, Salt Lake City, UT 84107, USA; viet.le@imail.org; 6 Intermountain Medical Center Heart Institute, Department of Biomedical Informatics, University of Utah, Salt Lake City, UT 84107, USA

**Keywords:** intermittent fasting, intermittent energy restriction, coronary artery disease, diabetes, trimethylamine *N*-oxide, metabolic analytes

## Abstract

Intermittent fasting (IF) has been connected with health benefits such as weight loss, lower risk of coronary artery disease (CAD) and diabetes, increased longevity, and improved quality of life. However, the mechanisms of these IF benefits in humans require further investigation. This study sought to elucidate some of these mechanisms through secondary analyses of the Fasting and ExprEssion of Longevity Genes during fOOD abstinence (FEELGOOD) trial, in which apparently healthy participants were randomized in a Latin square design to a 24-h water-only fast and a 24-h ad libitum fed day. Two pathways were investigated, with trimethylamine *N*-oxide (TMAO) levels measured due to their association with elevated risk of CAD, along with conductance of a broad panel of metabolic analytes. Measurements were made at baseline, at the end of the fasting day, and at the end of the fed day. A fasting mean of 14.3 ng in TMAO was found versus the baseline mean of 27.1 ng with *p* = 0.019, although TMAO levels returned to baseline on refeeding. Further, acute alterations in levels of proline, tyrosine, galactitol, and urea plasma levels were observed along with changes in 24 other metabolites during the fasting period. These acute changes reveal short-term mechanisms which, with consistent repeated episodes of IF, may lead to improved health and reduced risk of CAD and diabetes.

## 1. Introduction

Dietary energy restriction through regular fasting has been associated with beneficial health improvements including reduced risk of coronary artery disease (CAD) and diabetes in observational studies of coronary angiography patients [1]. Mechanistically, decreases in glucose concentrations, insulin resistance, cholesterol, weight, and white fat tissue have been observed in fasting animal studies [2]. Some mice models showed improvements in glucose regulation and in the development of neuronal resistance to injury [3]. Furthermore, animal studies found that fasting increases longevity [4]. Together, these findings suggest a powerful potential tool in fasting that may improve metabolic, cardiovascular, and cognitive health, among possible other effects. Whether these results translate to humans is uncertain, both because of the biological differences of the more complex human physiology and because of the inability to specify or tightly control fasting regimens among humans compared to animals in a laboratory setting.

Among humans, water-only fasting performed for no more than 24-h at a time has substantiated that important physiologic changes occur due to such energy restriction [5]. Trials of repeated episodes of fasting have been undertaken with a variety of regimens examined and a few high-quality trials with parallel control arms now complete. Alternate-day fasting, in which a person abstains from food for 24-h (consuming only one 500-calorie meal in the middle of the day) has demonstrated reduced weight, decreased waist circumference, and improved insulin resistance in obese and prediabetic participants [6,7]. Two-day per week fasting (the so-called 5:2 diet) is reported to improve hemoglobin A1c by an amount equivalent to a standard weight loss diet and to reduce weight, waist circumference, and body fat [8,9]. The mechanisms for these improvements in cardiometabolic risk factors along with the potential long-term improvements in longevity and reduced risk of CAD and diabetes are currently incompletely understood and require further investigation.

Understanding the physiological changes during a fasting episode can help advance the understanding of the mechanisms underlying any benefits anticipated from repeated fasting. Trimethylamine *N*-oxide (TMAO) is an amine oxide produced in humans by intestinal microbiota from excess trimethylamine (TMA), an intermediate of choline metabolism [10]. TMAO has been linked to cardiovascular disease in humans, where high plasma levels of TMAO are associated with inhibiting cholesterol transport, activating inflammation in adipose tissue, impairing glucose tolerance, and inhibiting insulin signaling in the liver [2,11,12]. Additionally, increased TMAO plasma levels in mice have been shown to enhance atherosclerosis [11]. Plasma levels of TMAO are affected by dietary intake and the current microbiome composition of the gastrointestinal tract [12].

Currently, there are no human data on the effects of intermittent fasting on TMAO levels or on a broad survey of metabolic analytes [13]. While investigation of such changes across multiple episodes would be a key to evaluating whether fasting can mollify risks related to TMAO or metabolites, whether fasting can cause changes to such parameters is needed to justify the investigation of long-term improvements. This study utilized stored samples from the Fasting and ExprEssion of Longevity Genes during fOOD abstinence (FEELGOOD) trial to evaluate the effect of fasting on TMAO and, secondarily, a panel of metabolite levels among apparently healthy individuals to determine if changes in these analytes may contribute to the benefits observed with fasting.

## 2. Materials and Methods

The FEELGOOD clinical cross-over trial was conducted over a two-day participation period and consisted of 30 participants with no chronic disease diagnoses [5]. Participants were randomized in a 1:1 manner using a Latin square design to a “fasting first” group or an initially not fasting group (see below). Controls for this trial were the participants themselves, which self-matched factors for gender, age, and body mass index (BMI), as well as other subject-specific potential confounders. The Intermountain Healthcare Institutional Review Board approved the FEELGOOD Trial, and all participants consented to participation, including for long-term sample storage and testing of new hypotheses in stored samples. The FEELGOOD Trial was registered at ClinicalTrials.gov prior to initiation of enrollment (identifier NCT01059760). Diet and daily caloric consumption of the participants were not evaluated either before or during the FEELGOOD Trial. The primary hypothesis of this new analysis of the FEELGOOD Trial was that TMAO levels are altered by a 24-h water-only fast. Secondary hypotheses were that 74 other metabolites are changed by a 24 h water-only fast. Multiple comparisons correction was used to identify factors that were significantly changed.

As previously reported, participant ages were 43.6 ± 13.5 years, with the youngest being 18 years old and the oldest 70 years [5]. Two-thirds of the study population were female. BMI averaged 27.8 ± 1.3 kg/m^2^. Other baseline characteristics have been previously published [5].

Individuals were included if they had not fasted more than 12 h at a time during the previous year, did not regularly limit calories by less than 80% of US Food and Drug Administration recommendations within the past two years, and did not regularly skip meals for dieting. To ensure participants were relatively healthy, individuals were not included in the trial if they had the following: myocardial infarction, stroke, immune system disorders or deficiency, peripheral vascular disease, active insulin administration, active cancer treatments, active immunosuppressive treatment, solid organ transplant within the year, and current or past smoking.

Participants were randomly assigned into one of two groups, each of which would complete 28 ± 4 h of water-only fasting and 28 ± 4 h of ad libitum eating (with 28 h ensuring that at least 24 h were reached). One group (i.e., fasting first) began with the water-only fast followed by the fed session. The other group began with the fed session first (i.e., initially not fasting), followed by the water-only session [5]. Samples were drawn from the participants before treatment, at the day one visit, and at the day two visit for each group.

In this new analysis of FEELGOOD biological samples, TMAO and 74 other metabolic analytes were measured including amino acids and fatty acids. These 74 metabolites were compromised of: lactic acid, pyruvate, glycerol, glyceric acid, citric acid, aconitate, isocitric acid, 2-ketoglutaric acid, succinic acid, fumaric acid, malic acid, 2-aminoadipic acid, lysine, valine, leucine, isoleucine, threonine, glycine, serine, alanine, glutamic acid, glutamine, proline, aspartic acid, asparagine, methionine, cysteine, phenylalanine, tyrosine, tryptophan, histidine, ornithine, phosphate, diphosphate, phosphoglycerol, 3-phosphoglycerate, fructose, galactose, glucose-6-phosphate, mannitol, sorbitol, galactitol, inositol, myoinositol phosphate, sucrose-trehalose, lauric acid, myristic acid, palmitelaidic acid, palmitic acid, linoleic acid, oleic acid, elaidic acid, stearic acid, arachidonic acid, 1-monooleoyglycerol, 1-monostearylglycerol, 2-monostearylglycerol, squalene, xanthine, hypoxanthine, uracil, adenosine-5’-monophosphate, erythrosine, erythrose-4-phosphate, tocopherol, B-alanine, 2-ketoisocaproic acid, gluconic acid, ascorbic acid, uric acid, and urea. Total cholesterol, glucose, and creatinine were also measured in this panel to provide a measure of quantitative validation of the panel because they had previously been tested using clinical diagnostics and analyzed for the effect of fasting [5].

Metabolic analytes were measured by gas chromatography-mass spectrometry (GC-MS). TMAO was measured via liquid chromatography-mass spectrometry (LC-MS) [14]. An internal standard d4-succinate was used to normalize for sample preparation and instrument drift for the 74 metabolic analytes. Absolute concentrations were not determined for the metabolites, but instead the area under the curve was used to obtain relative measures of concentrations compared to the standard. Determination of TMAO was calculated using an internal standard d9-TMAO with an external calibration curve drawn using this standard for the determination of absolute TMAO concentrations measured in nanograms.

A paired *t*-test was conducted to compare the results across fasting and fed days. The fasting change was calculated as the difference between each value of TMAO or the other metabolic analytes at the end of the fasting day minus the beginning value 24-h earlier. The beginning value for the fasting change was defined as the baseline value for those randomized to fast first and the 24-h fed value for those randomized to being fed the first day. The change during the fed day was defined as the value of the analyte at the end of the fed day minus the beginning value for that day, with the beginning value defined similarly to that of the fasting day (i.e., the fasting day end value used as the beginning value for those randomized to fast the first day, and the baseline value used as the beginning value for those randomized to have the fed day first). For the analysis of TMAO, statistical significance was defined as *p* ≤ 0.05. For the other 74 metabolic analytes, a *p*-value of *p* ≤ 0.05 was defined as suggestively significant requiring validation and the Bonferroni-corrected significance was achieved at *p* ≤ 0.000676 to correct for the elevated probability of false positive findings due to testing of multiple hypotheses. SPSS v.23.0 (IBM SPSS Statistics, Chicago, IL, USA) was used to perform all analyses with two-sided tests of hypothesis used for all analyses.

## 3. Results

**Figure 1:**
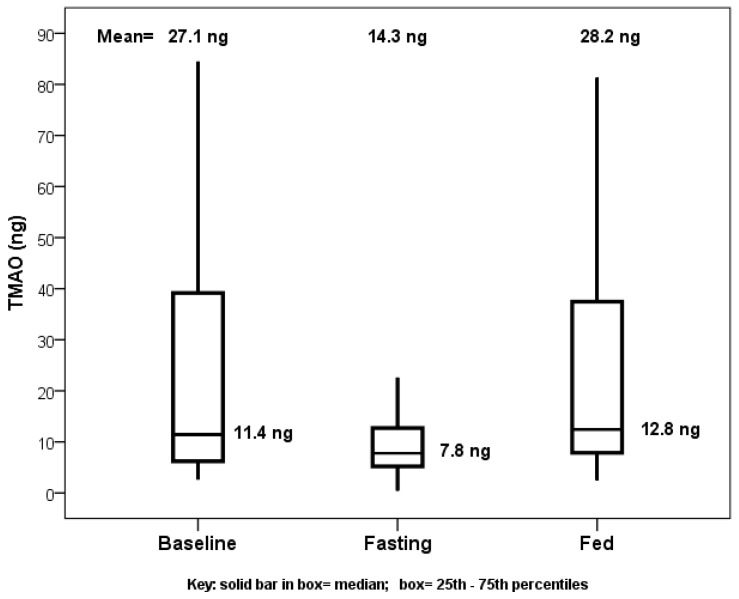
Boxplot of trimethylamine *N*-oxide (TMAO) levels when fed at baseline upon completion of fasting day and upon completion of the fed day using 24-h values of TMAO. Comparing baseline to fasting (*p* = 0.049) and fed to fasting (*p* = 0.019) showed significance, but the comparison of baseline to fed (*p* = 0.93) did not show significance.

**Figure 2:**
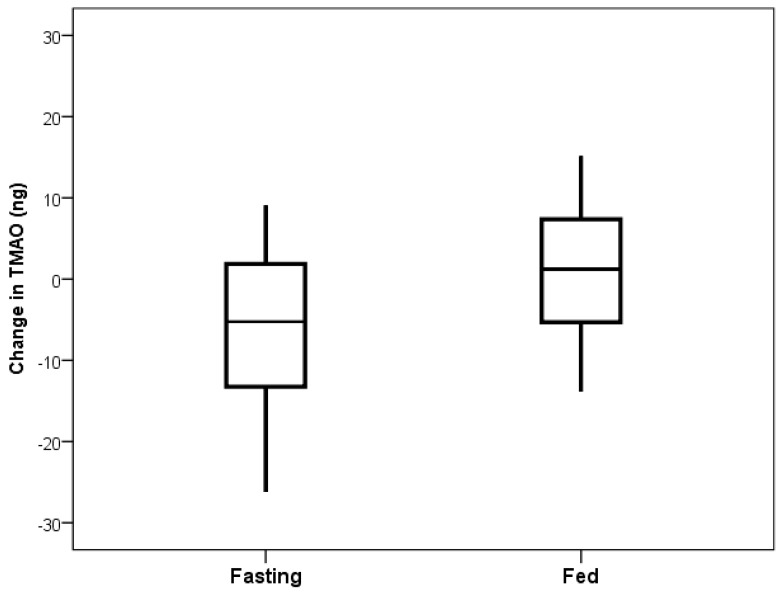
Boxplot comparing TMAO change on the 24-h fasting day versus the 24-h fed day. Fasting mean was −10.8 ng and fed mean was +2.6 ng (*p* = 0.25).

Baseline characteristics were previously reported [5]. Comparing TMAO baseline values or values from the end of the fed day to those at the end of the fasting day showed significance, with no difference between baseline and fed values (Figure 1). The primary comparison of the TMAO level changes from the beginning to the end of the fasting day with the changes from the beginning to the end of the fed day showed a decrease in TMAO levels when fasting (mean change: −10.8 ng) and an increase when fed (mean change: +2.6 ng), but this was not significant (*p* = 0.23, Figure 2).

**Figure 3:**
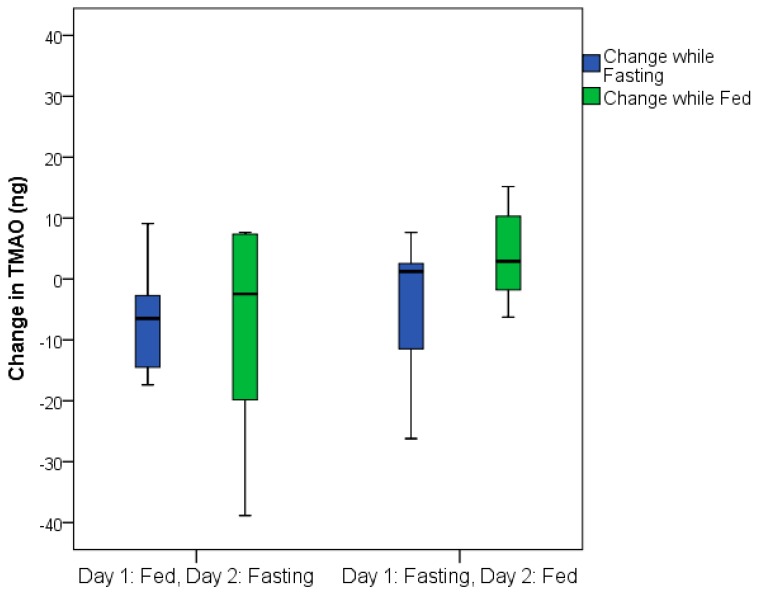
Boxplots displaying the similarity of TMAO changes for the group that fasted on the first day compared to those who fasted on the second day for the 24-h fasting period (*p* = 0.41) and for the 24-h fed day (*p* = 0.29).

**Figure 4:**
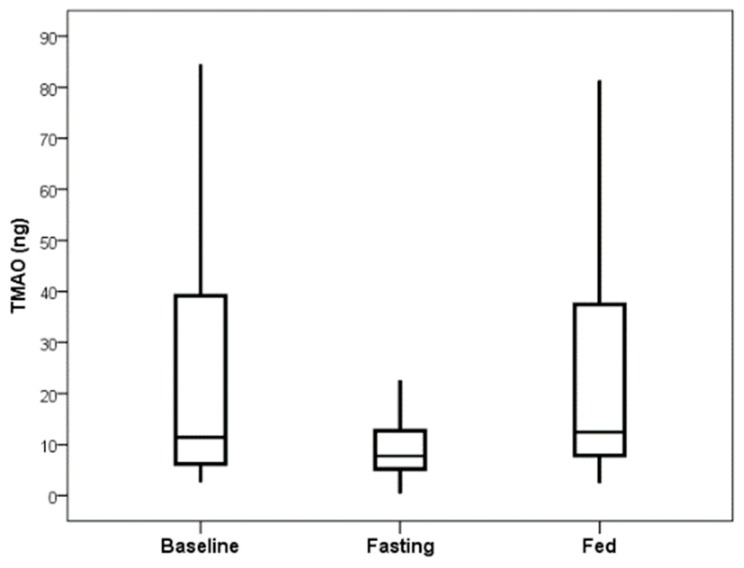
TMAO levels returned to the baseline level at 48 h after study start for those randomized to the group that fasted first, with baseline mean of 22.5 ng TMAO and 48-h mean (24 h after fasting completion) of 28.8 ng (*p* = 0.55).

The total TMAO change was similar in both the group that fasted on the first day and the group that was fed on the first day (Figure 3). TMAO levels returned to baseline values within 24 h following fasting completion (i.e., 24 h after feeding resumed) for the group that fasted on the first day (Figure 4).

Of the 74 other metabolic analytes measured, 30 achieved a suggestively significant *p*-value of *p* ≤ 0.05. These analytes include 16 amino acids and six fatty acids (Table 1). Another approach for evaluating the multiple comparisons is to examine the error rate for a test of 74 hypotheses, which is four false positives and in this case leaves 26 of the 30 remaining analytes being true positives, although which of the 26 are true positives is not discoverable. Values for 27 of the 30 adjusted analytes returned to baseline value within 24 h after the end of the fasting period in the 16 subjects of the fasting-first group, not unlike the trend in TMAO values. Three of the 30 adjusted metabolite levels remained depressed compared to baseline, even 24 h after completion of the 24-h fasting period: pyruvate with *p* = 0.014, glutamic acid with *p* = 0.025, and tryptophan with *p* = 0.028.

**Figure 5:**
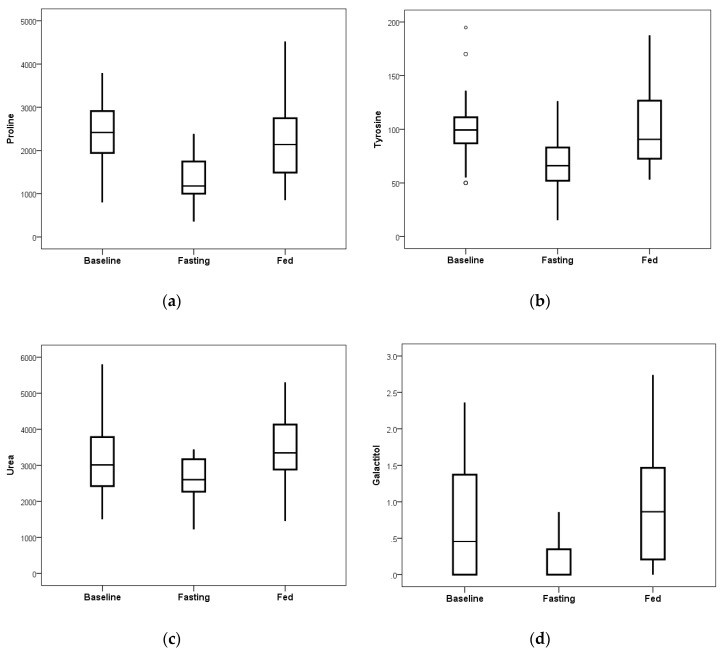
After 24 h of the fasting intervention, reductions were noted in proline, tyrosine, urea, and galactitol as compared to the observed changes on the ad libitum fed day that were statistically significant when corrected for the 74 comparisons. (**a**) Proline achieved *p* = 0.00002. (**b**) Tyrosine achieved *p* = 0.00033. (**c**) Urea achieved *p* = 0.00034. (**d**) Galactitol achieved *p* = 0.00058. Displayed values are relative measures of each metabolite compared to the internal d4-succinate standard. The bottom of the box is the 25^th^ percentile and the top is the 75^th^ percentile of data, while the whiskers extend up to 1.5 times beyond the height of the box (if data points exist within that interval). Open circles represent outliers that are more than 3-fold greater or less than the height of the box (see panel b where most baseline values were tightly clustered in a small interval but three values were quite divergent).

Blood plasma levels of proline, tyrosine, galactitol, and urea showed substantial reduction due to the fasting intervention as compared to the observed changes on the ad libitum fed day (Figure 5). Calculated *p*-values for each analyte were *p* = 0.00002 for proline, *p* = 0.00033 for tyrosine, *p* = 0.00034 for urea, and *p* = 0.00058 for galactitol, showing significance in the amount of reduction.

## 4. Discussion

Intermittent fasting is associated with improved cardiovascular function, cardiovascular resilience, and even endurance performance [15,16]. Intermittent fasting-related decreases in resting heart rate, blood pressure, LDL cholesterol, weight, waist circumference, white adipose tissue, HbA1c, inflammation, and risk of cardiovascular disease have been previously observed [2,6,7,8,16]. These decreases have been detected alongside increases in longevity, insulin sensitivity, and HDL levels [4,8]. Additionally, CAD risk has been connected with changes in metabolic analyte surveys including branched chain amino acids [17,18,19].

Although various benefits of intermittent fasting have been seen in animal studies, including increased longevity and reduced weight, the mechanisms behind the non-weight loss health benefits have not yet been fully evaluated, and some mechanisms may remain to be determined that require further investigation in humans [4]. Previously, we reported from the FEELGOOD Trial that human growth hormone was dramatically elevated during fasting, red blood cell count and hemoglobin were increased without hemoconcentration, and circulating cholesterol levels were significantly increased due to 24 h of water-only fasting [5]. The results of this new set of tests using stored samples from FEELGOOD showed that fasting significantly reduced TMAO and significantly altered proline, tyrosine, urea, and galactitol blood plasma levels.

TMAO is a novel and exciting biomarker of cardiovascular disease in humans that arises from intestinal microbiota [10,11]. TMAO may impair metabolism and is linked to a pro-inflammatory response [12]. Due to its connection to dietary intake, TMAO is also a good candidate to be affected by intermittent fasting, and its change highlights the possibility that fasting may also beneficially alter the microbiome at least during caloric desistance, if not for a more extended period of time after the end of a fasting episode. If the changes are only temporary during fasting, as the results herein suggest, this may have several results. First, fasting may provide an environment in which a change of diet may more rapidly and beneficially alter the microbiome away from the pro-atherogenic composition that is associated with higher TMAO. This would require additional intervention beyond the fasting regimen studied here, potentially requiring a new diet that would resist the development of the microbiota that increase TMAO levels.

During the fasting period of the study, TMAO levels were substantially decreased during the fasting day compared to the fed day. However, during the fed period, TMAO production rapidly returned back to concentrations similar to baseline levels. The change score for the fasting day compared to the fed day was not statistically significant, but the improvement in TMAO was tightly controlled, with substantially less variation when fasting than at the fed or baseline measurement. This TMAO reduction followed by a resumption of baseline levels is likely a result of elimination from the body of the substrate needed to produce TMAO [11]. Without this substrate being replenished through the diet, reduction of TMAO was observed. When this substrate was replenished during the fed stage, TMAO production appeared to be recommenced. This lack of substrate may be as simple as the elimination of matter from the gastrointestinal tract during fasting and could lead to a period of physiological rest from the cardiovascular risk associated with TMAO.

Alternatively, the bacteria that produce TMAO may have been eliminated from the gastrointestinal tract during fasting. While more difficult to conceive of how this may occur in a 24-h period, this also would not explain the rapid resumption of TMAO produced within the day of feeding. Likely, recolonization of the bacteria within the intestine should take longer than the one day observed for TMAO production to resume [10].

Regardless, these results indicate that regular intermittent fasting may alter TMAO levels, which may decrease the chance of CAD development for at least the duration of fasting [3]. This observed TMAO change may have important clinical significance as one potential mechanism for a cardiovascular health benefit from intermittent fasting that could occur periodically due to a 24-h fast [10]. The long-term effects of fasting on TMAO should be researched in future studies to determine the full implications for human health. Further, these data suggest that studies are needed of the impact of fasting on the overall human microbiome and what such changes to the constituents of the microbiome may mean for possible reductions in chronic disease risk by fasting.

In addition to TMAO plasma reduction, acute decreases in plasma levels of amino acids and acute increases in circulating levels of fatty acids were found. The lower plasma levels of amino acids were a class effect, suggesting a stark decrease in protein metabolism during fasting and potentially an increase in protein synthesis. Most notably, the amino acids proline and tyrosine were observed to be reduced following the 24-h water-only fasting intervention. Increased levels of these metabolites are connected to higher blood pressure, more depression, and greater insulin resistance [20,21]. The changes observed here, may thus explain some of the potential health and longevity benefits from fasting, with decreased levels of proline and tyrosine being associated with diminished symptoms of depression, lower resistance to insulin, and improved cognitive function [20]. Further evaluation of the impact of amino acid changes during and after fasting is required to determine the possible mechanisms of health benefits from fasting.

Urea plasma levels were reduced as well at the completion of the fasting period. This effect of fasting on urea is to be expected given the decreases in circulating amino acids and change in protein metabolism/synthesis. Urea is typically elevated during protein metabolism as the deamination of amino acids and excretion of nitrogen occur. Further, urea is involved in the natriuresis of fasting through nitrogen excretion [21]. This may aid in lowering blood pressure, a common cardiovascular risk factor [22].

As a class, fatty acids were increased during the fasting period, with six fatty acids achieving suggestive statistical significance (with *p* < 0.05). While fatty acids tend to be chronically elevated in obesity and insulin resistance, the acute changes measured here are indicative of short-term use of fatty acids for energy rather than as increases in disease risk [23]. Ketone levels were not measured in this study, but the elevated circulating fatty acids suggests that ketosis was achieved during the 24-h fast. Beyond direct use for energy, fatty acids in the plasma may also be used to synthesize biologically significant factors like triglycerides and ketone bodies. Unfortunately, no individual fatty acid change achieved the multiple comparisons-corrected level of significance (*p* ≤ 0.000676). Further evaluation of the release of fatty acids during periodic fasting is indicated. Also, the investigation of the extent to which ketosis occurs during a water-only fast is required, including the timing of the transition to ketosis and—given the interest today in time-restricted feeding—the level of effect of ketosis at each hour after the body has switched to ketosis (and up to 24 h or longer of fasting).

Finally, a reduction in galactitol plasma levels was observed due to fasting. Galactitol is a sugar alcohol with potentially harmful neurotoxic and metabotoxic effects in high concentrations that is, along with glucose, one of the products of the metabolism of galactose [24]. Galactitol production may be restricted during fasting as circulating glucose is reduced and fatty acids are utilized as the primary energy source. While this decrease in galactitol may be a marker of the body entering ketosis, which may lead to a decrease in body fat and insulin resistance overall, it is unclear whether other mechanisms are at play with respect to galactitol that may contribute to reduced chronic disease or increased longevity.

Future investigations should also evaluate the three metabolites that all remained decreased from baseline even after the fed day among participants who fasted the first day. These three factors were pyruvate (the end product of glycolysis), glutamic acid (an amino acid and excitatory neurotransmitter that is involved in memory, learning, and other cognitive effects) [25], and tryptophan (an amino acid and neurotransmitter that is converted to niacin in the liver, utilizing vitamin B6 [26], which plays a role in the synthesis of the neurotransmitter serotonin) [27]. While not significant after correction for multiple comparisons, these results should be investigated further in future studies, especially because of the connection to cognitive function, to determine whether these parameters remain altered well after a fasting period has ended and for how long they remain changed. The four factors that were changed the most during fasting, and the other 23 metabolites that had suggestively significant associations, did not remain changed once eating resumed. The final values 24 h after eating recommenced were not different from baseline for proline (*p* = 0.61), tyrosine (*p* = 0.23), urea (*p* = 0.90), or galactitol (*p* = 0.34).

A 24-h water-only fasting intervention in apparently healthy individuals who were enrolled in the FEELGOOD Trial influenced changes in TMAO and many of 74 other metabolites analyzed in this study. Health benefits such as decreased insulin resistance, increased lipid metabolism, decreased weight and body fat, lower levels of depression, and increased cognitive performance may be the result of such changes. The mechanisms and results of those changes should be investigated further in longer-term studies with repeated episodes of intermittent fasting that do not necessarily focus on weight loss.

## Figures and Tables

**Table 1:** Of the 74 metabolic analytes measured, 30 achieved suggestive significance at *p* ≤ 0.05, including four that had *p* < 0.000676 (Figure 5). Six fatty acids increased through fasting and 16 amino acids were reduced through fasting. Values for each metabolite are relative measures based on the internal standard as determined from the area under the curve.

Metabolic Analyte	Baseline	Fasting Change	Fed Change	*p*-Value
Pyruvate	39.2 ± 15.5	−13.7 ± 21.7	3.7 ± 20.5	0.031
Glycerol	403 ± 118	155 ± 289	−64 ± 282	0.046
Lysine	563 ± 215	−204 ± 340	109 ± 360	0.023
Valine	2270 ± 579	−497 ± 983	364 ± 954	0.017
Isoleucine	658 ± 182	−201 ± 343	131 ± 384	0.019
Threonine	107 ± 33	−31.8 ± 38.9	19.5 ± 46.0	0.003
Glycine	75.0 ± 23.6	−12.0 ± 17.2	4.3 ± 18.7	0.012
Alanine	172 ± 39	−56.4 ± 69.0	30.8 ± 64.9	0.0009
Glutamic Acid	29.7 ± 15.4	−12.9 ± 22.2	5.4 ± 21.4	0.030
Glutamine	228 ± 50	−39.2 ± 65.8	17.7 ± 55.5	0.009
Proline	2386 ± 751	−1068 ± 932	494 ± 950	0.00002
Aspartic Acid	43.7 ± 19.3	−16.1 ± 22.0	7.8 ± 18.5	0.002
Methionine	130 ± 45	−50.1 ± 61.7	30.4 ± 67.5	0.002
Phenylalanine	251 ± 59	−62.1 ± 92.0	25.5 ± 86.0	0.0111
Tyrosine	101 ± 31	−43.3 ± 43.6	22.7 ± 46.9	0.0003
Tryptophan	535 ± 194	−187 ± 221	70 ± 240	0.005
Histidine	46.8 ± 23.4	−14.1 ± 25.6	6.4 ± 27.9	0.006
Ornithine	16.2 ± 6.4	−5.66 ± 7.08	2.19 ± 8.14	0.006
Phosphoglycerol	44.9 ± 31.1	−23.9 ± 27.0	6.6 ± 32.5	0.005
Fructose	166 ± 113	−118 ± 182	61 ± 202	0.010
Galactitol	0.67 ± 0.71	−0.72 ± 0.87	0.68 ± 1.47	0.00058
Inositol	225 ± 71	-58.2 ± 66.0	29.4 ± 75.4	0.001
Myoinositol Phosphate	117 ± 34	-36.9 ± 53.7	7.5 ± 57.1	0.030
Lauric Acid	21.3 ± 11.9	12.3 ± 27.4	−8.8 ± 24.0	0.034
Myristic Acid	116 ± 64	71.1 ± 119	−27.1 ± 94.5	0.012
Palmitelaidic Acid	12.9 ± 11.7	36.2 ± 48.8	−10.5 ± 46.2	0.009
Linoleic Acid	167 ± 77	64.6 ± 147	−19.1 ± 99.8	0.049
Oleic Acid	264 ± 174	418 ± 534	−118 ± 481	0.004
Elaidic Acid	20.8 ± 17.2	32.8 ± 40.9	−7.5 ± 38.2	0.004
Urea	3410 ± 1228	−786 ± 1083	456 ± 880	0.0003
